# *Centella asiatica* Attenuates Mitochondrial Dysfunction and Oxidative Stress in A*β*-Exposed Hippocampal Neurons

**DOI:** 10.1155/2017/7023091

**Published:** 2017-08-13

**Authors:** Nora E. Gray, Jonathan A. Zweig, Donald G. Matthews, Maya Caruso, Joseph F. Quinn, Amala Soumyanath

**Affiliations:** ^1^Department of Neurology, Oregon Health and Science University, Portland, OR 97239, USA; ^2^Department of Cell, Developmental and Cancer Biology, Oregon Health and Science University, Portland, OR 97239, USA; ^3^Department of Neurology and Parkinson's Disease Research Education and Clinical Care Center (PADRECC), Portland Veterans Affairs Medical Center, Portland, OR 97239, USA

## Abstract

*Centella asiatica* has been used for centuries to enhance memory. We have previously shown that a water extract of *Centella asiatica* (CAW) protects against the deleterious effects of amyloid-*β* (A*β*) in neuroblastoma cells and attenuates A*β*-induced cognitive deficits in mice. Yet, the neuroprotective mechanism of CAW has yet to be thoroughly explored in neurons from these animals. This study investigates the effects of CAW on neuronal metabolism and oxidative stress in isolated A*β*-expressing neurons. Hippocampal neurons from amyloid precursor protein overexpressing Tg2576 mice and wild-type (WT) littermates were treated with CAW. In both genotypes, CAW increased the expression of antioxidant response genes which attenuated the A*β*-induced elevations in reactive oxygen species (ROS) and lipid peroxidation in Tg2576 neurons. CAW also improved mitochondrial function in both genotypes and increased the expression of electron transport chain enzymes and mitochondrial labeling, suggesting an increase in mitochondrial content. These data show that CAW protects against mitochondrial dysfunction and oxidative stress in A*β*-exposed hippocampal neurons which could contribute to the beneficial effects of the extract observed in vivo. Since CAW also improved mitochondrial function in the absence of A*β*, these results suggest a broader utility for other conditions where neuronal mitochondrial dysfunction occurs.

## 1. Introduction

Alzheimer's disease (AD) is the most common form dementia affecting an estimated 5.4 million people in the United States alone [1]. Despite continued increase in the prevalence of AD, effective therapies remain limited owing, at least in part, to an incomplete understanding of the underlying biology of the disease. In AD patients, the accumulation of *β*-amyloid (A*β*) plaques and neurofibrillary tangles is accompanied by synaptic dysfunction, cell death, and severe cognitive impairment [2, 3]. There is a growing evidence that mitochondrial dysfunction and oxidative stress contribute to these deleterious physiological changes [4]. Alterations in mitochondrial bioenergetics as well as mitochondrial mass and enzyme expression in the brain are thought to precede, or even induce, cognitive decline in AD [5–8]. Increased oxidative stress is likewise considered to be an early event in the brains of AD patients [9, 10]. These abnormalities have been observed in many in vitro and in vivo AD model systems as well [11–13].

The plant *Centella asiatica* (L.) Urban (Apiaceae) has been used traditionally in Chinese and Ayurvedic medicine to improve cognitive function [14]. Extracts of the plant have been shown to have neuroprotective and cognitive-enhancing effects in a variety of in vitro and in vivo models of neurodegenerative disease and in the setting of neurotoxic insults [15–17]. Our own lab has reported that a water extract of *Centella asiatica* (CAW) attenuates cognitive impairment in the Tg2576 mouse model of A*β* accumulation [18]. These mice express a mutant form of human amyloid precursor protein (APP) leading to age-dependent A*β* plaques in the hippocampus and cortex and concomitant learning and memory deficits. Two weeks of treatment with CAW in the drinking water normalized the behavioral deficits in the Tg2576 animals [18]. We have observed similar cognitive-enhancing effects in healthy-aged wild-type (WT) animals, and this enhancement was accompanied by an increase in the expression of mitochondrial and antioxidant response genes in the brains of those animals [19].

Our lab has recapitulated many of the beneficial effects of CAW using in vitro models. We have observed that CAW treatment protects against A*β*-induced cell death, mitochondrial dysfunction, and oxidative stress in human neuroblastoma cells [20, 21]. However, neuroblastoma cells may not accurately reflect the bioenergetics of neurons [22], and the effects of the extract on isolated neurons have yet to be thoroughly investigated. This study aims to address this gap in understanding by examining the effects of CAW on mitochondrial function and oxidative stress in hippocampal neurons isolated from Tg2576 mice and their WT littermates.

## 2. Materials and Methods

### 2.1. Aqueous Extract of *Centella asiatica*

Dried *Centella asiatica* was purchased (Oregon's Wild Harvest, GOT-03193c-OHQ01), and its identity was confirmed by comparing its thin layer chromatographic profile with that reported in the literature [23] and the *Centella asiatica* samples used in our previous studies [18]. The water extract of *Centella asiatica* (CAW) was prepared by refluxing *Centella asiatica* (160 g) with water (2000 mL) for 2 hours, filtering the solution, and freeze drying to yield a powder (~16–21 g). Isolated neurons were treated with CAW at a concentration of 50 *μ*g/mL for 7 days.

The percent content of constituent triterpene compounds in CAW was assessed by HPLC coupled to UV detection (LC-UV) as previously described [20]. The levels of each of the four major triterpenes (asiatic acid, asiaticoside, madecassic acid, and madecassoside) were found to be similar to our previously published values [20, 24].

### 2.2. Culture of Primary Hippocampal Neurons

Embryonic Tg2576 mice and their wild type (WT) littermates were used to generate primary neuronal cultures. The Tg2576 line expresses the human APPswe double mutation (K670N-M671L) under the control of the hamster prion promoter [25, 26], resulting in an accumulation of A*β*_1–42_ in the brain and the development of age-dependent A*β* plaques. Primary neurons from these animals display metabolic abnormalities [27] and a dystrophic phenotype [28]. Breeding pairs of Tg2576 mice were raised in an in-house facility at OHSU. All procedures were conducted in accordance with the NIH Guidelines for the Care and Use of Laboratory Animals and were approved by the institutional Animal Care and Use Committee of OHSU and the VA Portland Medical Center.

Hippocampal neurons were isolated from embryonic mice, based on the methods of Kaech and Banker [29]. Briefly, embryos were harvested at 18 days of gestation from anesthetized females. The animals were genotyped by PCR using DNA extracted from tail samples taken after dissection of the hippocampi. Hippocampi were dissected, gently minced, and trypsinized to generate suspensions of dispersed neurons, which were then plated on to poly-l-lysine-coated dishes in MEM medium (GIBCO/Life Technologies), 5% FBS (Atlanta Biologicals), and 0.6% glucose (Sigma-Aldrich). After 4 hr, the medium was removed and replaced with Neurobasal Medium supplemented with 1x GlutaMAX (GIBCO/Life Technologies) and 1x GS21 (MTI-GlobalStem). After five days in vitro, cells were treated with 1 *μ*M cytosine *β*-D-arabinofuranoside hydrochloride (AraC; Sigma-Aldrich) and CAW (50 *μ*g/mL). All analyses were performed at 7 days in vitro.

### 2.3. Intracellular ROS Quantification

ROS levels were determined using OxiSelect Intracellular ROS Assay kit (Cell biolabs) as per the manufacturer's instructions. Neurons were grown on 96-well plates at a density of 50,000 cells/well. On day 7, cells were incubated with the fluorogenic probe for 1 h at 37°C prior to measurement. Values were normalized to protein content determined by a bicinchoninic acid (BCA) protein assay as per the manufacturer's instructions (Pierce Biotechnology).

### 2.4. Lipid Peroxidation Measurement

The degree of neuronal lipid peroxidation was assessed by quantifying intracellular maldondialdehyde levels using a thiobarbituric acid reactive products (TBARs) assay kit (Cayman Chemicals). Neurons were grown on 12-well plates at a density of 250,000 cells/well. On day 7, cells were harvested in phosphate buffered saline, sonicated, and TBARs measured as per the manufacturer's instructions. Values were normalized to protein content determined by BCA as above.

### 2.5. Analysis of Mitochondrial Function

Mitochondrial function was assessed using the Seahorse Bioscience XF24 Extracellular Flux Analyzer. Neurons were plated on Seahorse XF culture plates (Seahorse Bioscience) at a density of 65,000 cells/well. On day 7, cells were rinsed in assay medium (pH 7.4) containing XF Base medium (Seahorse Bioscience), 5.5 mM glucose, and 1 mM sodium-pyruvate. Cells remained in assay medium 1 h at 37°C in a non-CO2 incubator prior to initializing the Seahorse24XF analysis. Using the MitoStress Kit as previously described [30], oxygen consumption rate (OCR) was measured under varying conditions. After three initial baseline measurements of OCR, the ATP synthase inhibitor oligomycin (1 *μ*m) was added and three subsequent measurements were taken. Next, an electron transport chain (ETC) accelerator, *p*-trifluoromethoxy carbonyl cyanide phenyl hydrazone (FCCP at 1.5 *μ*m), was added, and after 3 measurements were taken, mitochondrial inhibitors rotenone (1 *μ*m) and antimycin (1 *μ*m) were added, and three final measurements were taken. Data was normalized to total DNA content, which was determined from each well using the CyQuant kit (Invitrogen) as per the manufacturer's instructions.

### 2.6. ATP and Protein Quantification

ATP was quantified using the ATP determination kit (Life Technologies), as per the manufacturer's instructions. Neurons were plated on 12-well plates at a density of 250,000 cells/well. On day 7, cells were lysed with 0.1% Triton X 100 in PBS and incubated with the reaction solution for 15 min at room temperature, prior to measurement. Values were normalized to total protein content, as determined by BCA as above.

### 2.7. Mitochondrial Number

Relative mitochondrial number was determined using MitoTracker Green dye (Invitrogen) as per the manufacturer's instructions. Briefly, cells were incubated with 100 nM dye for 25 minutes, rinsed in fresh media, and fluorescence was quantified using a Victor 3 fluorescent plate reader (Perkin Elmer). Fluorescence was normalized to total protein content, determined by BCA as above.

### 2.8. Gene Expression

Neurons were plated on 12-well plates at a density of 200,000 cells/well. On day 7, total RNA was extracted using Tri-Reagent (Molecular Research Center). RNA was reverse transcribed with the Superscript III First Strand Synthesis kit (Invitrogen) to generate cDNA, as per the manufacturer's instructions. Relative gene expression was determined using TaqMan Gene Expression Master Mix (Invitrogen) and commercially available TaqMan primers (Invitrogen) for nuclear factor (erythroid-derived 2)-like 2 (NRF2, also called NFE2L2), NAD(P)H dehydrogenase-quinone oxidoreductase 1 (NQO1), glutamate-cysteine ligase, catalytic subunit (GCLC), heme oxygenase 1 (HMOX1), mitochondrially encoded NADH dehydrogenase 1 (Mt-ND1), mitochondrially encoded ATP synthase 6 (Mt-ATP6), mitochondrially encoded cytochrome c oxidase 1 (Mt-CO1), mitochondrially encoded cytochrome B (Mt-CYB), and glyceraldehyde-3-phosphate dehydrogenase (GAPDH). Quantitative PCR (qPCR) was performed on a StepOne Plus Machine (Applied Biosystems) and analyzed using the delta-delta Ct method.

### 2.9. Statistics

Statistical significance was determined using Bonferroni post hoc tests and one-way analysis of variance with appropriate *t*-tests. Significance was defined as *p* ≤ 0.05. Analyses were performed using the Excel or GraphPad Prism 6.

## 3. Results

### 3.1. CAW Activates Endogenous Antioxidant Response Pathway and Attenuates Oxidative Stress in A*β*-Exposed Hippocampal Neurons

**Figure 1:**
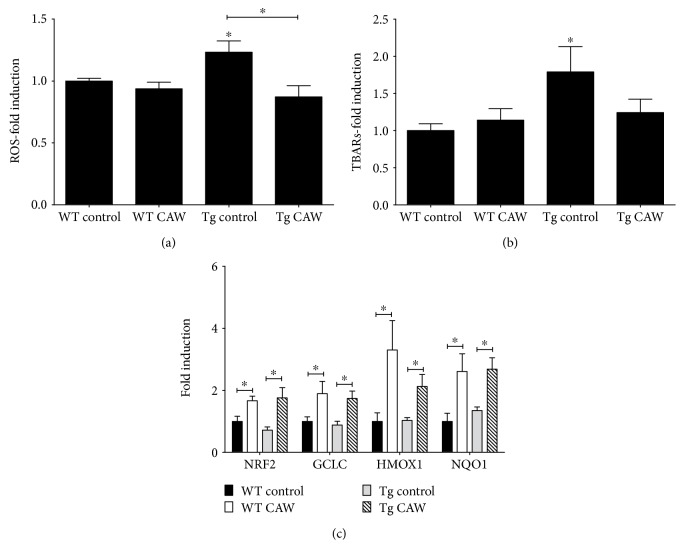
CAW attenuates A*β*-induced oxidative stress and activates antioxidant response pathway in Tg2576 and WT hippocampal neurons. (a) Levels of intracellular reactive oxygen species (ROS) were elevated in Tg2576 neurons, and this increase was attenuated by CAW treatment (50 *μ*g/mL; *n* = 16 − 20). (b) CAW treatment reduced the increase in lipid peroxidation observed in Tg2576 neurons, as quantified by TBARs (*n* = 11–13). (c) CAW induced the expression of NRF2 and its target antioxidant genes in both Tg2576 and WT neurons (*n* = 9–12). ^∗^*p* < 0.05 compared to WT control unless otherwise indicated.

Tg2576 animals overexpress human APPswe double mutation and develop age-dependent A*β* plaques in the hippocampus and cortex [25, 26]. As previously reported, cultured neurons from Tg2576 develop degenerative phenotypes due to their overproduction of A*β* [27, 28]. We found that after seven days in culture, Tg2576 neurons showed a significant increase in intracellular ROS relative to WT neurons (Figure 1(a)). This increase was accompanied by a similar increase in oxidative damage to lipids (Figure 1(b)). Two days of treatment with CAW (50 *μ*g/mL) restored the levels of ROS and lipid peroxidation to WT control levels (Figures 1(a) and 1(b)). CAW treatment had no effect on either ROS or lipid peroxidation in WT neurons (Figures 1(a) and 1(b)). CAW treatment also robustly increased the expression of NRF2 and its target antioxidant defense genes in both WT and Tg2576 neurons (Figure 1(c)).

### 3.2. CAW Improves Mitochondrial Function in Hippocampal Neurons

**Figure 2:**
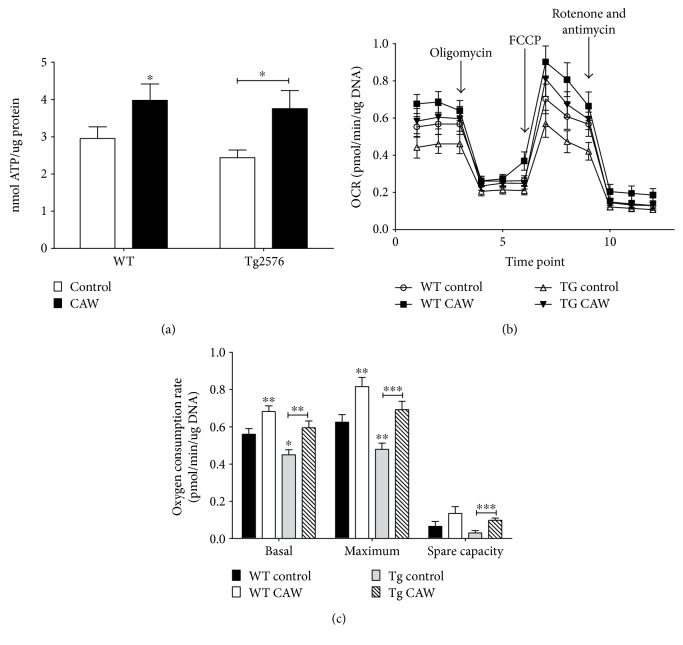
CAW improves mitochondrial function in Tg2576 and WT hippocampal neurons. (a) CAW treatment (50 *μ*g/mL) increased ATP content in neurons from both genotypes (*n* = 6). (b) CAW attenuated bioenergetic deficits in Tg2576 neurons. Oxygen consumption rate (OCR) was significantly reduced in Tg2576 cells at baseline and after FCCP stimulation. CAW attenuated this decrease under both conditions. In WT cells, CAW treatment also significantly increased basal- and FCCP-stimulated OCR. There were no differences in OCR between genotypes or treatment groups after either oligomycin or rotenone and antimycin treatment (*n* = 11–14). (c) Tg2576 neurons exhibited diminished basal and maximal OCR relative to WT neurons, and CAW treatment attenuated these decreases. CAW also increased spare capacity in Tg2576 neurons (*n* = 11–14). ^∗^*p* < 0.05, ^∗∗^*p* < 0.01, and ^∗∗∗^*p* < 0.001 compared to WT control unless otherwise indicated.

Two days of CAW treatment (50 *μ*g/mL) significantly increased ATP content in both WT and Tg2576 hippocampal neurons (Figure 2(a)). The bioenergetic profile of hippocampal neurons treated with CAW for two days was determined using the Seahorse XF Analyzer (Figure 2(b)). Under basal conditions, Tg2576 neurons exhibited reduced oxygen consumption rate (OCR) compared to WT neurons. Treatment with CAW normalized the basal OCR of the Tg2576 neurons to WT control levels. In WT neurons, CAW treatment also enhanced basal OCR levels. There were no differences in OCR between treatment groups following the addition of oligomycin or rotenone and antimycin indicating normal response to these inhibitors. In contrast, maximal respiratory rate, following FCCP treatment, was reduced in Tg2576 neurons, and this impairment was attenuated by CAW treatment. CAW also increased maximal respiration in WT neurons.

By averaging the three measurements taken sequentially at baseline and after FCCP stimulation, average basal and maximal OCRs were calculated. Tg2576 neurons had reduced basal and maximal OCRs, and CAW treatment attenuated these decreases (Figure 2(c)). CAW treatment in WT neurons significantly increased both basal and maximal OCR as well. The difference between the maximal OCR and the basal OCR is the spare capacity of the cell and reflects the amount of extra ATP that can be generated in response to a sudden increase in energy demand. CAW treatment significantly increased spare capacity in Tg2576 neurons (Figure 2(c)).

### 3.3. CAW Increased Mitochondrial Content in Hippocampal Neurons

**Figure 3:**
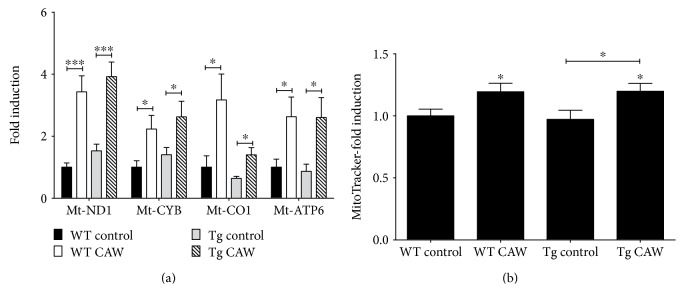
CAW increases mitochondrial content in Tg2576 and WT hippocampal neurons. (a) CAW treatment (50 *μ*g/mL) increased the expression of genes encoding enzymes in the electron transport chain (ETC) in both Tg2576 and WT neurons (*n* = 9–12). (b) CAW increased mitochondrial labeling by the fluorescent dye MitoTracker Green relative to controls in both Tg2576 and WT neurons (*n* = 8–10). ^∗^*p* < 0.05, ^∗∗∗^*p* < 0.001 compared to WT control unless otherwise indicated.

The expression of the mitochondrial DNA-derived genes Mt-ND1, Mt-CYB, Mt-CO1, and Mt-ATP6, which encode proteins in complexes I, III, IV, and V of the ETC, respectively, was evaluated in isolated hippocampal neurons. CAW treatment coordinately increased the expression of all four genes in both the Tg2576 and WT neurons consistent with an increase in mitochondrial content (Figure 3(a)). The fluorescent dye MitoTracker was used to determine relative mitochondrial number. CAW treatment significantly increased mitochondrial content in both WT and Tg2576 neurons (Figure 3(b)).

## 4. Discussion

The neuroprotective effects of CAW have been well-documented both in vitro and in vitro [16, 17, 31–34]. Our lab has previously reported that CAW improves cognitive function in both healthy mice as well as those that accumulate A*β* plaques [18, 19] and protects against the toxic effects of A*β* in neuroblastoma cells [20, 21]. In this study, we characterize the mitochondrial and antioxidant effects of CAW in A*β*-exposed hippocampal neurons.

We found that CAW activated the endogenous antioxidant response pathways by increasing the expression of NRF2 and its target genes in neurons isolated from Tg2576 embryos that overexpress human APPswe, a mutant form of APP that leads to A*β* accumulation in human patients [25]. Concomitant with this activation was an attenuation of the increased levels of intracellular ROS and lipid peroxidation that were apparent in the Tg2576 neurons. These effects are consistent with the previous reports of the antioxidant effects of *Centella asiatica* in both in vitro and in vivo models of A*β* exposure [21, 35, 36]. It is the A*β* in the culture media that is likely responsible in the increased neuronal oxidative stress. It has been shown previously that conditioned media from Tg2576 neuronal cultures induces aberrant calcium signaling in WT neurons while immunodepletion of A*β* in that conditioned media completely blocks this effect in WT neurons [28]. Although future experiments are necessary to definitively confirm that A*β* immunodepletion prevents the increase in ROS and lipid peroxidation, reported here, we anticipate that it will since calcium dyshomeostasis is known to participate in the increased free radical generation and mitochondrial dysfunction seen in response to A*β* exposure [37, 38].

There was a similar increase in antioxidant gene expression in WT neurons in response to CAW but no resulting change in the levels of either ROS or TBARs. Because they also serve as critical signaling molecules, ROS levels are tightly regulated in healthy cells so it is not surprising that there was no reduction observed in WT neurons. Notably, while increased levels of ROS and lipid peroxidation were observed in the A*β* overproducing Tg2576 neurons relative to WT, there were no differences in antioxidant gene expression between the genotypes. This is in contrast to our previous finding in neuroblastoma cells where A*β* exposure itself resulted in increased expression of NRF2 and its target genes [21]. One possible explanation could be that there was not sufficient A*β* accumulation during the time that the primary neurons were in culture to induce the expression of these genes. Many of the phenotypes associated with the Tg2576 neurons, such as the dystrophic morphology, do not manifest until several weeks in culture [28]. It is interesting though that there was sufficient A*β* accumulation to significantly increase the ROS and TBARs in these cells.

CAW also attenuated mitochondrial dysfunction in Tg2576 neurons. These A*β*-expressing neurons exhibited reduced basal and maximal respiration as well as spare capacity, and CAW restored the OCRs back to the wild type neuron levels. CAW also increased basal and maximal respiration in the absence of A*β*. Similarly, ATP levels were increased in both WT and Tg2576 neurons although no differences were observed between genotypes. The fact that bioenergetic impairments in oxygen consumption rates could be detected in Tg2576 while there were no differences in ATP content is likely due to the increased sensitivity of the Seahorse XF platform. Taken together, these findings support previous evidence of mitoprotective effect of *Centella asiatica* in animal models [39] as well as in vitro [21].

In addition to altering neuronal mitochondrial activity, CAW also increased the expression of mitochondrial enzymes and appeared to increase mitochondrial content, suggesting a possible effect on mitochondrial biogenesis. While the increase in mitochondrial gene expression is consistent with what we have previously observed in the brains of aged WT animals treated with CAW [19], to our knowledge, there are no other reports of effects of *Centella asiatica* on mitochondrial content. Asiatic acid, a triterpene found in *Centella asiatica*, has been shown to induce the expression of peroxisome proliferator-activated receptor gamma coactivator 1-alpha (PGC1*α*), the so-called master regulator of mitochondrial biogenesis, in neuroblastoma cells [40]. It is possible that this could explain how CAW is affecting biogenesis as well because although CAW does not contain detectable levels of asiatic acid it does contain the glycosidic form of the compound, asiaticoside, which can be metabolized into asiatic acid [20]. We are currently evaluating the effects of CAW on the expression of PGC1*α* and other regulators of mitochondrial biogenesis. It is also possible that the increase in mitochondrial gene expression does not reflect a change in biogenesis at all but rather a shift in mitochondrial dynamics. Expression of fission- and fusion-related proteins has been shown to be altered in Tg2576 neurons [27, 41]. Electron microscopy studies are underway in our lab to quantify the number and size of mitochondria in these neurons in response to CAW.

Future studies are necessary to confirm the effects of CAW on mitochondrial dysfunction and oxidative stress in vivo. Additionally, it remains to be seen what role these mitochondrial and antioxidant effects play in the cognitive-enhancing properties of the extract that we have observed in Tg2576 and aged WT animals. Other studies have shown that modulating antioxidant and mitochondrial pathways results in neuroprotection and enhanced cognitive performance in rodents [42–44] so it is possible that these same mechanisms contribute to the cognitive-enhancing effects of CAW we have observed in our mouse models of aging and AD. Since mitochondrial dysfunction and oxidative stress accompany cognitive impairment in many other neurodegenerative diseases, as well, the therapeutic potential of CAW may extend beyond AD.
